# Impact of the first COVID-19 epidemic wave in a large French network of nursing homes: a cross-sectional study

**DOI:** 10.1186/s12877-023-04078-8

**Published:** 2023-07-03

**Authors:** Fabrice Mbalayen, Sarah Mir, Valentine de l’Estoile, Aude Letty, Solenn Le Bruchec, Manon Pondjikli, Elise Seringe, Gilles Berrut, Fariba Kabirian, Marie-Anne Fourrier, Didier Armaingaud, Loïc Josseran, Elisabeth Delarocque-Astagneau, Sylvain Gautier

**Affiliations:** 1 University Department Public Health, Prevention, Observation, Territories – UFR Simone Veil – Santé, Université Versailles Saint-Quentin-en-Yvelines, Montigny-le-Bretonneux, France; 2 Département hospitalier d’épidémiologie et de santé publique, hôpital Raymond-Poincaré, Groupe hospitalier universitaire Université Paris-Saclay, Assistance publique- Hôpitaux de Paris, Garches, France; 3 Korian Foundation for the Ageing Well, Korian, Paris, SA France; 4 Gérontopôle Autonomie Longévité Pays de la Loire, Nantes, 44200 France; 5 Centre d’appui pour la prévention des infections associées aux soins - CPias Île-de-France, Paris, France; 6 Centre Hospitalier Universitaire de Nantes, Pôle Hospitalo-Universitaire de Gérontologie Clinique, Nantes, France; 7 Korian SA, Medical, Ethics and Quality Department, Paris, France; 8 Centre de recherche en épidémiologie et santé des populations, UMR 1018, Université de Versailles-Saint-Quentin-en-Yvelines, Université Paris-Saclay, Montigny-Le-Bretonneux, France

**Keywords:** COVID-19, Mortality, Nursing home, Elderly, First wave

## Abstract

**Background:**

Nursing homes (NHs) have been particularly affected by COVID-19. The aim of this study is to estimate the burden of COVID-19 and to investigate factors associated with mortality during the first epidemic wave in a large French NHs network.

**Methods:**

An observational cross-sectional study was conducted in September-October 2020. 290 NHs were asked to complete an online questionnaire covering the first epidemic wave on facilities and resident characteristics, number of suspected/confirmed COVID-19 deaths, and preventive/control measures taken at the facility level. Data were crosschecked using routinely collected administrative data on the facilities. The statistical unit of the study was the NH. Overall COVID-19 mortality rate was estimated. Factors associated with COVID-19 mortality were investigated using a multivariable multinomial logistic regression. The outcome was classified in 3 categories: “no COVID-19 death in a given NH”, occurrence of an “episode of concern” (at least 10% of the residents died from COVID-19), occurrence of a “moderate episode” (deaths of COVID-19, less than 10% of the residents).

**Results:**

Of the 192 (66%) participating NHs, 28 (15%) were classified as having an “episode of concern”. In the multinomial logistic regression, moderate epidemic magnitude in the NHs county (adjusted OR = 9.3; 95%CI=[2.6–33.3]), high number of healthcare and housekeeping staff (aOR = 3.7 [1.2–11.4]) and presence of an Alzheimer’s unit (aOR = 0.2 [0.07–0.7]) were significantly associated with an “episode of concern”.

**Conclusions:**

We found a significant association between the occurrence of an “episode of concern” in a NH and some of its organizational characteristics and the epidemic magnitude in the area. These results can be used to improve the epidemic preparedness of NHs, particularly regarding the organization of NHs in small units with dedicated staff.

**Brief summary:**

Factors associated with COVID-19 mortality and preventive measures taken in nursing homes in France during the first epidemic wave.

**Supplementary Information:**

The online version contains supplementary material available at 10.1186/s12877-023-04078-8.

## Background

In January 2020, the first cases of severe acute respiratory syndrome coronavirus 2 (SARS-CoV-2) infection were identified in France [1]. The rapidly increasing number of hospitalisations and intensive care unit admissions triggered the decision of a national lockdown by the French government on March 16th. The first epidemic wave was particularly intense and the lockdown lasted for almost two months [2]. From the beginning of the pandemic it was found that age and various pre-existing comorbidities such as cardiovascular disease, diabetes, and respiratory diseases were associated with an increased risk of severe cases of COVID-19 or death, pointing out older adults’ vulnerability [3, 4]. In May 2020, a summary note from the United Nations reported a mortality rate five times the average rate for people aged 80 or older [5]. More than 95% of COVID-19 deaths in Europe occurred in people aged 60 or older. In France, the French national institute for statistical and economic studies (INSEE) outlined a first wave of death in March-April 2020 with a 31% excess of all-caused deaths for 70 years and older compared to the same period in 2019 [6]. In addition, excess of death was higher in Paris area, east and north of France.

Nursing homes (NHs) have been particularly affected by COVID-19. As documented COVID-19 deaths in NHs represented 30–60% of all COVID-19 deaths in many European countries [7]. Indeed, NHs residents were more likely to be exposed to COVID-19 due to community life and to develop a severe form due to their advanced age and comorbidities. A large study, using hospitalisation and reimbursement data from the public and national health data base, confirmed the high burden of deaths among NHs residents, as they account for half of the total excess of deaths in France during the first wave of the COVID-19 pandemic [8]. Although numerous protection measures were implemented, it was estimated that three quarters of French NHs had at least one resident infected in 2020 [9]. One out of five NHs reported at least 10 deaths or a 10% death rate among their residents. To investigate factors associated with COVID-19 morbidity and mortality, a retrospective cohort study was conducted in 15,038 American NHs with long-stay residents during the first wave. The results showed that the risk of SARS-CoV-2 infection was associated with the local magnitude of the epidemic, while risk of hospitalization and death after SARS-CoV-2 infection was mostly associated with individual resident characteristics [10]. In a study conducted among 600 NHs in Ontario, Canada, from March to May 2020, crowding, expressed as the mean number of residents per bedroom and bathroom, was associated with an increased incidence of infection and mortality [11]. In Europe, few studies were conducted in the early phase of the pandemic on the factors associated with higher mortality in NHs. A study, including 1,145 residents of 27 French NHs from a private group, conducted after the first wave of COVID-19, explored individual risk factors for infection [12]. The most dependent residents, living in a protected unit due to behavioral disorders had significantly higher rates of infection. Another study showed that among 3 French NHs, the one not linked to a hospital had more COVID-19 cases and more deaths compared to the two others [13]. A study in which the characteristics of 57 NHs, located in North-Eastern Italy, were investigated as potential risk factors for COVID-19 infection, found that the risk of COVID-19 outbreaks was associated with the geographical location of the NHs but not to structural factors and standard infection prevention and control measures implemented [14].

Despite an apparently abundant literature on COVID-19 in NHs, most of the studies investigated the infections at an individual level (risk of infection, risk of death…) rather than at the level of the NH on a population-based approach. The few studies using this approach were mainly conducted in North America. In Europe, they remained scarce, limited by the sample size or mainly focused on COVID-19 incidence rather than on the mortality.

Therefore, the objectives of the present study were to estimate the burden of COVID-19 and to investigate factors associated with mortality during the first epidemic wave in a large French private NHs network. Factors considered included structural and organizational characteristics of the NHs, residents’ characteristics, and the local epidemic magnitude.

## Methods

This observational cross-sectional study was conducted during the first wave of the epidemic in a French private NHs network composed of 290 structures, corresponding to 22,540 residents, i.e. 4% of the NHs’ residents in France during the first epidemic wave.

### Data sources

Data were collected between September 14th 2020 and October 27th 2020 via an online questionnaire distributed exclusively to the directors of the NHs and covering the period from March to July 2020. We followed the recommendations for developing high-quality online questionnaires [15]. All these informed data were completed and validated using administrative data, routinely collected, available for each NH of the network over the study period. The cumulative number of deaths per NH was also cross-checked with the number of deaths reported to the public health authorities according to a standardized definition [16]. In order to characterize the magnitude of the epidemic in each NH area, we used excess all ages mortality data during the first wave produced by INSEE [17]. Thus, three levels were considered: low (category of reference for analysis), moderate and high, corresponding to counties with a mean excess of death of 5.2%, 44.5% and 110.5% respectively. In order to illustrate the magnitude of the epidemic according to the French metropolitan regions, we used data on mortality in the elderly population (≥ 80 y.o.) (as number of COVID-19 death recorded over the elderly population) during the first wave from the French National Mortality Database (INSERM CepiDC) provided by Santé publique France, the French public health agency.

### Definitions

COVID-19 deaths were defined as deaths occurring during the first epidemic wave, confirmed by a PCR or suspected by a physician. The recorded COVID-19 deaths corresponded to the COVID-19 deaths of the nursing home residents and included both COVID-19 deaths that occurred within the facility and COVID-19 deaths that occurred in the hospital during a hospitalization. The impact of COVID-19 in terms of mortality in NHs was classified with a three-modality indicator, considering the 10% threshold of mortality usually used by French authorities to describe COVID-19 mortality in NHs [9]. An episode was defined as “of concern” when at least 10% of the residents of a given NH died from COVID-19 during the first epidemic wave. An episode was defined as “moderate” when there were deaths from COVID-19 in the NH but less than 10% of the residents died from it. The third modality was “no deaths”. These mortality indicators were built to put forward, at the level of each NHs, the excess mortality in relation to the local epidemic context as well as to its main characteristics. The factors tested allowed us to study different determinants: external (epidemic wave, etc.) and internal to the NHs (presence of a coordinating physician, Alzheimer’s unit, audit of practices, etc.) and specific to its residents (mean age, dependency level, etc.). Healthcare and housekeeping staff represented staff in contact with the residents. It is counted as a number of person and not in full-time equivalent. The coordinating physician is the healthcare professional in charge of developing and monitoring the NHs’ care project, the medical evaluation of residents and the management of the care team. The NHs residents’ level of dependency is evaluated by the “Groupe Iso-Ressource moyen pondéré” (GMP), indicator which allows an evaluation of residents’ dependency on the NH scale. The score of dependency of a resident is based on the evaluation of eight measures of disability and two additional measures of intellectual coherence and orientation [18]. The higher the GMP score, the greater the average level of residents’ dependency. It ranges from 70 to 1,000. The NHs residents’ care needs are evaluated by another indicator, the “Pathos moyen pondéré” (PMP), which synthetized the level of care required to manage all the pathologies of the residents of a NH. It is calculated using the “PATHOS” tool [19], which allocates points according to observed clinical situations (the higher the PMP score, the greater the need for care).

### Statistical analysis

The statistical unit of the study was the NH. COVID-19 attributed, and all-causes mortality rates were calculated as the number of COVID-19 deaths observed per hundred residents, for the former, and as the number of deaths from all causes observed per hundred residents for the second. Quantitative variables were converted in two-categorical variables, based on their median for the entire network (N = 290) of NHs.

Numbers and proportions were used to describe qualitative variables. For COVID-19 and all-causes mortality rates, 95% confidence interval of the mean were calculated. The primary outcome was the occurrence of an “episode of concern” within the NH during the first epidemic wave. Univariable and multivariable multinomial logistic regression were performed to investigate factors associated with the presence of an episode of concern. We calculated odds-ratio (OR) and adjusted odds-ratio (aOR) from the multinomial logistic regression model to estimate occurrences for all NHs. Factors associated with an episode of concern with a p-value ≤ 20% or considered epidemiologically relevant (as well as mean age and dependency score) and not correlated with each other, were included in the multivariable multinomial logistic regression model. Backward stepwise regression using AIC (Akaike criterion information) was used to elaborate the final model.

All analysis were performed on available data. Analyses were conducted using the free software *R*, version 3.6.2. The database, which does not contain any individual data (only data concerning nursing homes and aggregated data on residents such as average age, dependency level, and number of deaths), has been declared to the CNIL, the French national commission for information technology and civil liberties, under the registration number N°2218181.

## Results

Of the 290 NHs of the network surveyed, 192 (66%) responded to the online questionnaire, corresponding to 15 307 residents in these NHs during the study period. The mean age of the residents (mean of the mean age per NH) was 88.1 years and varied between 77 and 92 years.

**Fig. 1:**
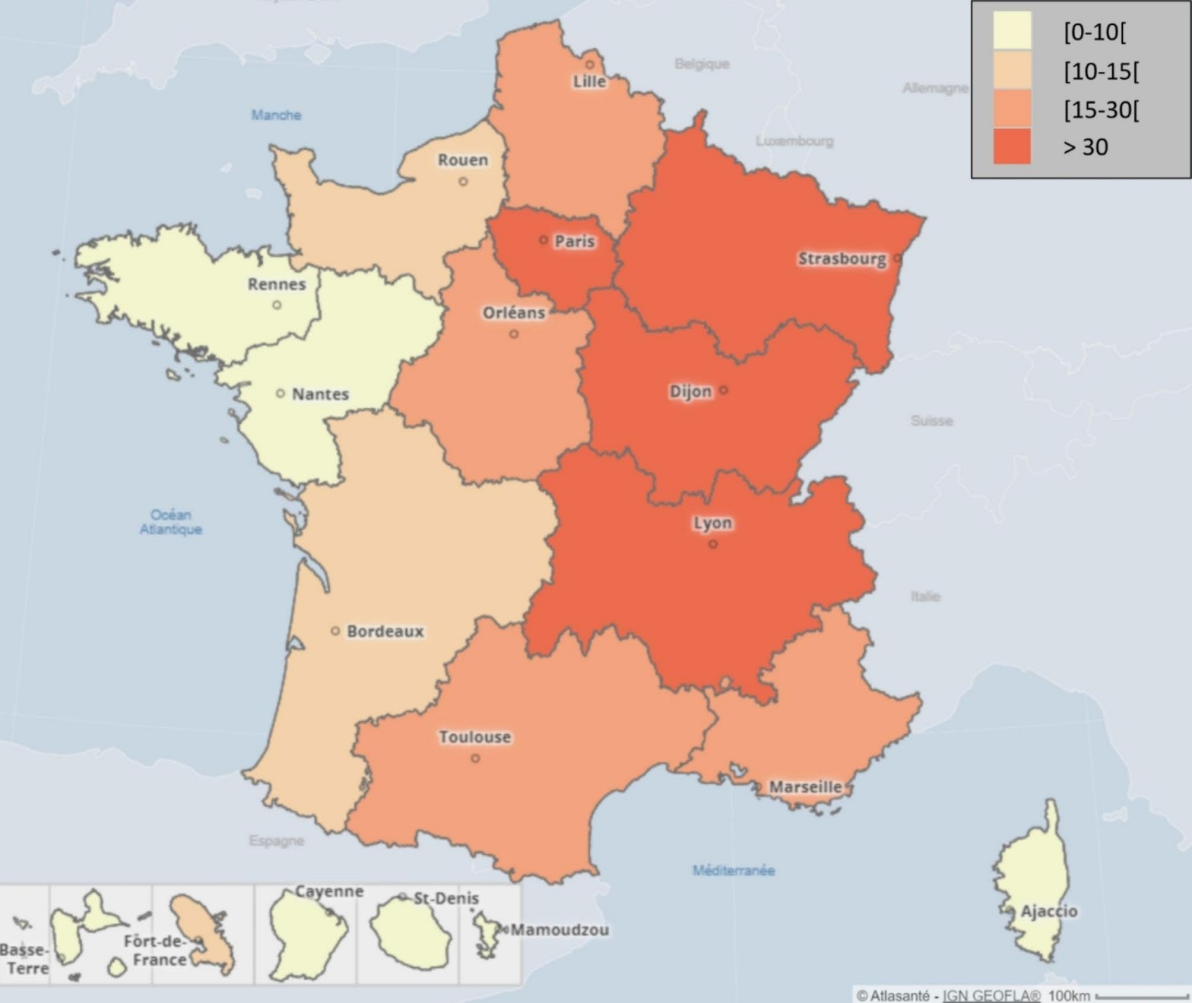
COVID-19 mortality of people aged 80 and over in France during the first wave, by region. In each region, the number of COVID-19 deaths during the first wave period (March-June 2020) was reported per 80 and over population of the region and presented as the number of COVID-19 deaths per 1,000 inhabitants. Data collected by Inserm CépiDC unit and processed by Santé publique France, the national public health agency. Map was created using the Cartos@nté tool

**Fig. 2:**
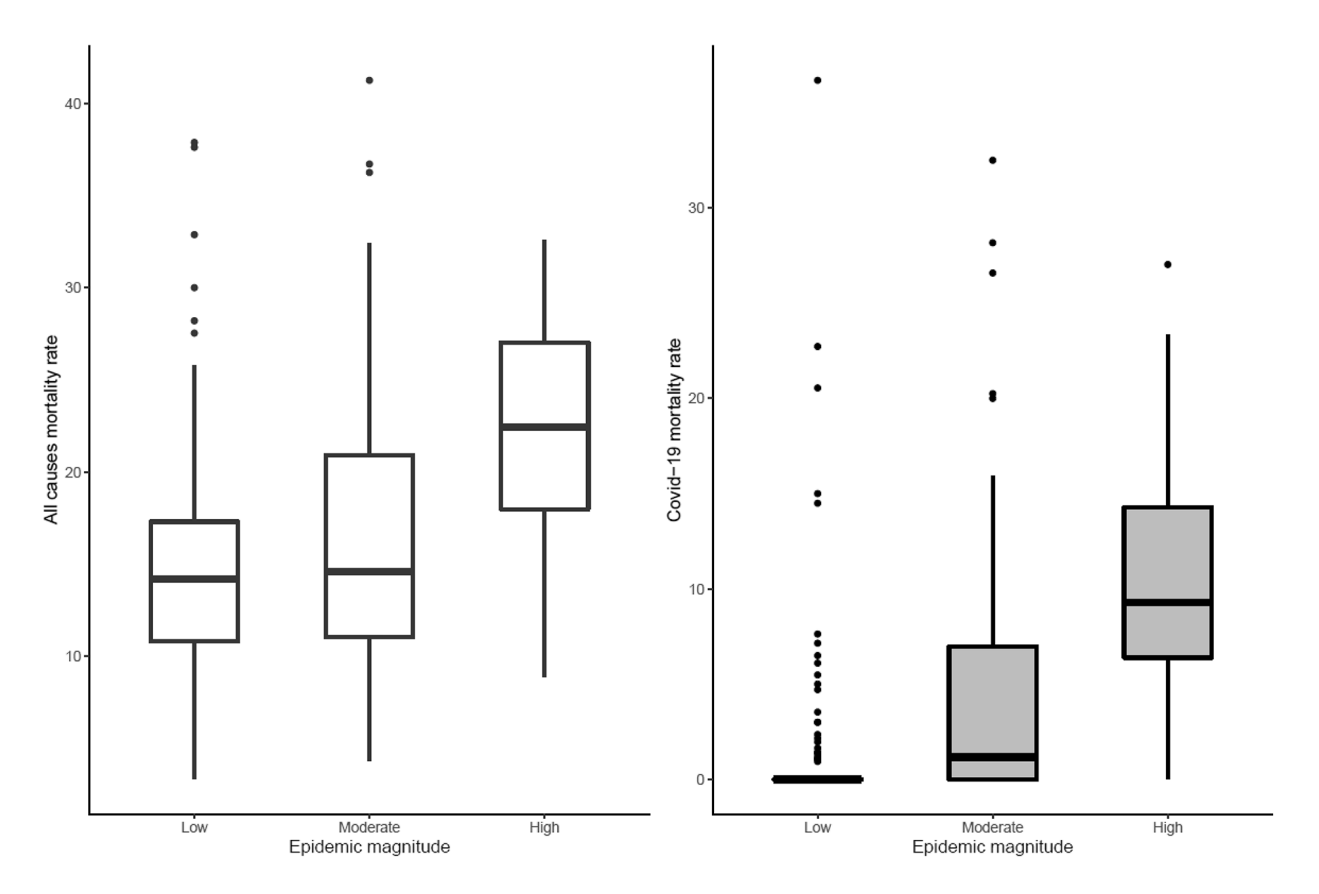
All-causes mortality and COVID-19 mortality rates in the nursing homes (N = 192) according to the magnitude of the epidemic. CluDe study, France, 2020 Reading notes: • COVID-19 mortality includes COVID-19 suspected or confirmed cases • Epidemic magnitude corresponds to 2020 excess of mortality compared to 2019 between March 1st to April 20th at the level of France counties (Monziols M. et al., 2020 (14)). “Low”, “moderate” and “high” categories correspond to counties with a mean excess of death of 5.2%, 44.5% and 110.5% respectively

Of the 192 participating NHs, 81% had a coordinating physician attached to their facility, 100% of the facilities had a hygiene coordinator, and 71% had an Alzheimer’s unit. The magnitude of the epidemic, measured as the COVID-19 mortality among people over 80 years old during the first wave by region, varied from 1 COVID-19 death per 1,000 inhabitants aged 80 and over in La Réunion region to 88 deaths per 1,000 inhabitants in the Île-de-France region (Paris area) (Fig. 1). 60% of the NHs were in counties where the magnitude of the epidemic was considered as low, 22% where it was considered as moderate and 17% where it was considered as high. Similar characteristics were observed between NHs of the network surveyed and our sample of 192 NHs (Table 1). The COVID-19 mortality varied according to the magnitude of the COVID-19 epidemic (Fig. 2).

**Table 1:** Main characteristics of the network of nursing homes, and of the participating nursing homes. CluDe study, France, 2020

		Network nursing homes N = 290	Participating nursing homes N = 192
		n/N^*^ (%)	n/N^*^ (%)
**High number of residents** ^**†**^		140/290 (48.2)	103/192 (53.6)
**Residents’ mean age over 88.5 years old** ^**†**^		140/290 (48.2)	92/192 (47.9)
**High dependency score** ^**†‡**^		140/290 (48.2)	95/192 (49.4)
**High need of care score** ^**†§**^		139/290 (47.9)	97/192 (50.5)
**High healthcare and housekeeping staff** ^†**^		130/290 (44.8)	96/192 (50.0)
**Coordinating physician (presence of)**		241/289 (83.3)	156/192 (81.2)
**Coordinating nurse (presence of)**		282/288 (97.5)	186/189 (98.4)
**Hygiene coordinator (presence of)**		290/290 (100.0)	192/192 (100.0)
**Alzheimer’s unit** ^**††**^ **(presence of)**		--	137/192 (71.3)
**Space for medical or nursing care** ^**††**^ **(presence of)**		--	142/192 (73.9)
**Physiotherapy space** ^**††**^ **(presence of)**		--	129/192 (67.1)
**“Snoezelen room” (presence of)**		179/289 (61.9)	119/191 (62.3)
**Epidemic magnitude** ^**‡‡**^	**Low**	177/290 (61.0)	116/192 (60.4)
	**Moderate**	67/290 (23.1)	43/192 (22.3)
	**High**	46/290 (15.8)	33/192 (17.1)

^*^ Missing and non-applicable data were subtracted from total nursing homes

^†^ Residents’ number, residents’ mean age, dependency score, need of care score and healthcare and housekeeping staff were dichotomized via the median split. Median values = 77, 88.5, 733, 228 and 49 respectively

^‡^ Dependency score is evaluated using the “Groupe Iso-Ressource moyen pondéré”, indicator which allows an evaluation of residents’ dependency at the nursing home scale

^§^ Need of care score is evaluated using the “Pathos moyen pondéré”, indicator which synthetized the level of care required to manage all the pathologies of the residents of a nursing home

^**^ Number of person and not full-time equivalent

^††^ Data only available for participating nursing homes

^‡‡^ Corresponds to 2020 excess of mortality compared to 2019 between March 1st to April 20th at the level of France counties (Monziols M. et al., 2020 (14)). “Low”, “moderate” and “high” categories correspond to counties with a mean excess of death of 5.2%, 44.5% and 110.5% respectively

More than 2/3 of hospitalization requests were met. The all-causes and COVID-19 mean mortality rates were 16.4% and 3.9% respectively. An ongoing outbreak in the surroundings was reported by 39% of the NHs. Of the 192 NHs, 53 (28%) were classified as having a “moderate episode” and 28 (15%) as having an “episode of concern” (Table 2).

**Table 2:** COVID-19 mortality and accepted hospitalization requests for COVID-19 among nursing homes. CluDe Study, France, 2020

N = 192	%	Confidence interval 95%
**Accepted hospitalization requests for COVID-19** ^*^	68.5	-
**All-causes mortality rate**	16.4	15.4–17.5
**COVID-19 mortality rate** ^**†**^	3.9	2.9–4.9
** N = 192**	**n (%)**	
**Moderate episode** ^**‡**^ **(presence of)**	53 (27.6)	
**Episode of concern** ^**§**^ **in the nursing home (presence of)**	28 (14.5)	
**COVID-19 clusters near the nursing homes**	75 (39.0)	

^*^ The total number of hospitalization requests for COVID-19 was 607 among 192 nursing homes

^†^ COVID-19 mortality includes COVID-19 suspected or confirmed cases

^‡^ An episode was considered as “moderate” when there were deaths from COVID-19 (suspected or confirmed) in the nursing home but less than 10%

^§^ An episode was considered as “of concern” when at least 10% of the residents of a given nursing home died from COVID-19 (suspected or confirmed)

Among the prevention and control measures taken by NHs, visitor bans were set up in 100% of the NHs, as well as residents’ isolation in all NHs where this measure could be applied. Dedicated units to COVID-19 patients, if the NHs had COVID-19 cases in this period, were carried out in 92% of the cases. For 64% of the NHs, external hygiene team acted as support. An audit of the hygiene practices among the healthcare staff was conducted in 73% of them (Table 3).

**Table 3:** Characteristics of the preventive measures taken by the nursing homes during the first wave of the COVID-19. CluDe Study, France, 2020

	Nursing homes N = 192	Epidemic magnitude^*^			
		Low N = 116	Moderate N = 43	High N = 33	
	n/*N*^**†**^ (%)	n/*N*^**†**^ (%)	n/*N*^**†**^ (%)	n/*N*^**†**^ (%)	
**Visitor bans**	192/192 (100.0)	116/116 (100.0)	43/43 (100.0)	33/33 (100.0)	
**Residents isolation**	189/192 (98.4)	114/116 (98.3)	42/43 (97.6)	33/33 (100.0)	
**Sectorization**	177/192 (92.1)	109/116 (93.9)	36/43 (83.7)	32/33 (96.9)	
**Dedicated COVID-19 unit** ^**‡**^	115/125 (92.0)	60/64 (93.7)	25/29 (86.2)	30/32 (93.7)	
***Day staff dedicated to this unit***^**§**^	*107/112 (95.5)*	*55/59 (93.2)*	*24/24 (100.0)*	*28/29 (96.5)*	
***Night staff dedicated to this unit***^**§**^	*85/112 (75.8)*	*39/56 (69.6)*	*19/25 (76.*0*)*	*27/30 (90.0)*	
**Support by an external hygiene team**	123/192 (64.0)	72/116 (62.0)	26/43 (60.4)	25/33 (75.7)	
**Standard precautions training in the previous 2 years**	187/192 (97.3)	111/116 (95.6)	43/43 (100.0)	33/33 (100.0)	
**Residents screening**	179/192 (93.2)	108/116 (93.1)	38/43 (88.3)	33/33 (100.0)	
**Staff screening**	184/192 (95.8)	110/116 (94.8)	41/43 (95.3)	33/33 (100.0)	
**Audit of practices**	141/192 (73.4)	87/116 (75.0)	27/43 (62.7)	27/33 (81.8)	

^*^ Corresponds to 2020 excess of mortality compared to 2019 between March 1st to April 20th at the level of France counties (Monziols M. et al., 2020 (14)). “Low”, “moderate” and “high” categories correspond to counties with a mean excess of death of 5.2%, 44.5% and 110.5% respectively

^†^ Missing and non-applicable data were subtracted from total nursing homes

^‡^ Sixty-seven nursing homes, including 52 for low, 14 for moderate and 1 for high, were not concerned by these items because they had no COVID-19 cases during the period

In univariate analysis, high number of residents (> 77) and high number of healthcare and housekeeping staff (> 49) were associated with an “episode of concern” (OR = 2.5, 95% Confidence Interval (CI) = [1.0–5.8] and OR = 2.3, 95%CI = [1.1–5.3] respectively). COVID-19 clusters near the NH were also associated with an “episode of concern” (OR = 2.3, 95%CI = [1.0–5.4]). In the same way, the magnitude of the epidemic was associated with an “episode of concern” when the excess of deaths in the county was considerate as moderate (OR = 7.6, 95%CI = [2.2–25.7]) and when it was high (OR = 135.0, 95%CI = [23.9–760.9]). Having an Alzheimer’s unit, and a coordinating physician were also associated with an “episode of concern” (OR = 0.3, 95%CI = [0.1–0.7] and OR = 0.4, 95%CI = [0.2–1.0] respectively) (Table 4).

**Table 4:** Factors associated with the presence of a moderate episode or an episode of concern in the nursing homes. Univariable multinomial logistic regression. CluDe study, France, 2020

N = 192		No deaths ^*^ N = 111 n (%)	Moderate episode^**†**^ N = 53 n (%)	Episode of concern^**‡**^ N = 28 n (%)		Moderate episode^**†**^ OR [CI95%]	p-value	Episode of concern^**‡**^ OR [CI95%]	p-value
**High number of residents** ^**§**^		47 (42.3)	38 (71.6)	18 (64.2)		3.5 [1.7–6.9]	< 0.001	2.5 [1.0-5.8]	0.04
**Residents’ mean age over 88.5** ^**§**^ **years old**		56 (50.4)	24 (45.2)	12 (42.8)		0.8 [0.4–1.5]	0.53	0.7 [0.3–1.7]	0.47
**High dependency score** ^§**^		59 (53.1)	23 (43.3)	13 (46.4)		0.7 [0.4–1.3]	0.24	0.8 [0.3–1.8]	0.52
**High need of care score** ^**§††**^		58 (52.2)	23 (43.3)	16 (57.1)		0.7 [0.3–1.3]	0.28	1.2 [0.5–2.8]	0.64
**High healthcare and housekeeping staff** ^**§‡‡**^		45 (40.5)	34 (64.1)	17 (60.7)		2.6 [1.3–5.2]	0.005	2.3 [1.1–5.3]	0.05
**Coordinating physician**		93 (83.7)	44 (83.0)	19 (67.8)		0.9 [0.4–2.3]	0.90	0.4 [0.2–1.0]	0.06
**Alzheimer unit**		85 (76.5)	38 (71.6)	14 (50.0)		0.8 [0.4–1.6]	0.50	0.3 [0.1–0.7]	0.007
**Audit of practices**		82 (73.8)	40 (75.4)	19 (67.9)		1.0 [0.5–2.3]	0.82	0.7 [0.3–1.8]	0.52
**COVID-19 clusters near the nursing home**		37 (33.3)	23 (43.3)	15 (53.5)		1.5 [0.8–3.0]	0.21	2.3 [1.0–5.4]	0.05
**Epidemic magnitude** ^**§§**^	**Low**	90 (81.0)	21 (39.6)	5 (17.8)		Ref.	-	Ref.	-
	**Moderate**	19 (17.1)	16 (30.1)	8 (28.5)		3.6 [1.6–8.2]	0.002	7.6 [2.2–25.7]	< 0.001
	**High**	2 (1.8)	16 (30.1)	15 (53.5)		34.3 [7.3–160.8]	< 0.001	135.0 [23.9–760.9]	< 0.001

^*^ Reference

^†^ An episode was considered as “moderate” when there were deaths from COVID-19 (suspected or confirmed) in the nursing home but less than 10%

^‡^ An episode was considered as “of concern” when at least 10% of the residents of a given nursing home died from COVID-19 (suspected or confirmed)

^§^ Residents’ number, residents’ mean age, dependency score, need of care score and healthcare and housekeeping staff were dichotomized via the median split. Median values = 77, 88.5, 733, 228 and 49 respectively

^**^ Dependency score is evaluated using the “Groupe Iso-Ressource moyen pondéré”, indicator which allows an evaluation of residents’ dependency at the nursing home scale

^††^ Need of care score is evaluated using the “Pathos moyen pondéré”, indicator which synthetized the level of care required to manage all the pathologies of the residents of a nursing home

^‡‡^ Number of person and not full-time equivalent

^§§^ Corresponds to 2020 excess of mortality compared to 2019 between March 1st to April 20th at the level of France counties (Monziols M. et al., 2020 (14)). “Low”, “moderate” and “high” categories correspond to counties with a mean excess of death of 5.2%, 44.5% and 110.5% respectively

In the multinomial logistic regression, the epidemic magnitude was significantly associated with an “episode of concern” with high (aOR = 118.0, 95%CI = [20.2–690.1]) and moderate (aOR = 9.3 [2.6–33.3]) excess of death compared to areas with low excess of death. High number of healthcare and housekeeping staff and presence of an Alzheimer’s unit were also significantly associated with an “episode of concern” (aOR = 3.7 [1.2–11.4] and aOR = 0.2 [0.07–0.7] respectively) (Table 5).

**Table 5:** Factors associated with the presence of a moderate episode or an episode of concern in the nursing homes. Multivariable multinomial logistic regression model, adjusted on the mean age and dependency score. CluDe study France, 2020

N = 192		No deaths^*^ N = 111 n (%)	Moderate episode^**†**^ N = 53 n (%)	Episode of concern^**‡**^ N = 28 n (%)		Moderate episode^**†**^ aOR [CI95%]	Episode of concern^**‡**^ aOR [CI95%]
**Alzheimer unit**		85 (76.5)	38 (71.6)	14 (50.0)		0.5 [0.2–1.4]	0.2 [0.07–0.7]
**High healthcare and housekeeping staff** ^§**^		45 (40.5)	34 (64.1)	17 (60.7)		3.0 [1.4–6.8]	3.7 [1.2–11.4]
**Epidemic magnitude** ^**††**^	**Low**	90 (81.0)	21 (39.6)	5 (17.8)		Reference	Reference
	**Moderate**	19 (17.1)	16 (30.1)	8 (28.5)		3.9 [1.6–9.1]	9.3 [2.6–33.3]
	**High**	2 (1.8)	16 (30.1)	15 (53.5)		32.2 [6.7–154.1]	118.0 [20.2–690.1]

^*^ Reference.

^†^ An episode was considered as “moderate” when there were deaths from COVID-19 (suspected or confirmed) in the nursing home but less than 10%.

^‡^ An episode was considered as “of concern” when at least 10% of the residents of a given nursing home died from COVID-19 (suspected or confirmed).

^§^ Healthcare and housekeeping staff were dichotomized via the median split. Median value = 49.

^**^ Number of person and not full-time equivalent.

^††^ Corresponds to 2020 excess of mortality compared to 2019 between March 1st to April 20th at the level of France counties (Monziols M. et al., 2020 (14)). “Low”, “moderate” and “high” categories correspond to counties with a mean excess of death of 5.2%, 44.5% and 110.5% respectively.

## Discussion

Our study, carried out in a large French network of NHs, allowed us to better understand the COVID-19 epidemic on this scale, with NHs constituting the statistical unit. It confirms the significant impact of the epidemic on the COVID-19 mortality of elderly people institutionalized in NHs in France even if more than half of the facilities did not report any COVID-19 deaths. The epidemic indeed concentrated in the early stages on certain metropolitan regions only where COVID-19 deaths occurred. A study conducted in England on 5,126 NHs also showed that during the first wave, half of the studied facilities did not observe any COVID-19 deaths [20]. Indeed, our results show a mortality rate of 3.9%, comparable to estimations reported in other studies, and the occurrence, in the NHs, of pejorative events, “moderate episode” or “episode of concern”, consistent with the observational data produced elsewhere [8], [9]. The analysis of these events reveals a significant association between their occurrence in a NH and, on the one hand, some organizational characteristics of the NH (i.e. the presence of an Alzheimer’s unit) and, on the other hand, the epidemic magnitude in the geographical area where the NH is located. The characteristics of the residents, such as mean age or dependency level, were not associated with the presence of an episode of concern when aggregated at the level of the NHs.

Thus, the epidemic magnitude where the NHs was located, represents a key explanatory factor in our results. We considered it at the county level (metropolitan France is divided into 96 counties), which seemed relevant because it was the smallest scale where epidemiological data were available for the first wave. Moreover, a simple typology at this scale could be mobilized to describe the epidemic magnitude. We found that the location most affected by COVID-19 (counties from Île-de-France and Grand-Est regions) had the highest mortality rates. This results are consistent with the epidemic dynamics in France at that time and are in line with other data describing the different levels of contamination and deaths according to NHs localization [21]. This differential impact is not specific to France. The first wave affected the different countries of the world in a very heterogeneous way, and the territorial impacts were as heterogeneous. In Europe this variability has been documented by a North-Eastern Italian study which found that the risk of COVID-19 outbreaks was associated with the geographical location of the NHs [14].

Beyond the local epidemic dynamics, in order to reduce the pandemic’s impact and protect their residents and staff, NHs took several preventive and control measures. Among them, wearing a mask was not always possible at the beginning of the pandemic, due to a worldwide shortage of personal protective equipment [22]. Some of these preventive and control measures were gradually implemented as hardware became available. This was particularly the case for massive screening, which was generally lacking on a global scale [23]. Some others could not be implemented uniformly because of NHs or residents’ specificities. For example, two NHs entirely composed of residents with neurodegenerative disorders decided, in agreement with local sanitary authority, not to isolate them in their rooms. The rate of the application of the measures was extremely high despite heterogeneities in the magnitude of the epidemic in the surrounding of the NHs. These local differences highlighted the capability that institutions had to complete the national or regional instructions, despite the constraining situation These results suggested the potential benefit of proportioning control measures, using a local scale instead of a national one, and the additional value of more local surveillance systems.

Moreover, the intrinsic characteristics of the NHs may have been decisive factors in limiting the entry of the virus into the structures and, if necessary, in increasing or slowing down the rate of contamination. In the literature, an American retrospective cohort study found that the structural and organizational characteristics of the NH facility were associated with the risk of SARS-CoV-2 infection, hospitalization and death [10]. As NH characteristics, we found that a higher number of healthcare and housekeeping staff was associated with COVID-19 death, as also found by McGarry et al. [24]. To better understand the full meaning of this association between high healthcare and housekeeping staff number and COVID-19 death, it would have been interesting to have qualitative data on workload, staff fatigue, well-being at work or mental health in this outbreak context [25].

At any rate, the staff may have played an important role in the spread of the epidemic within the NHs, at an early period when testing was limited. Despite the national lockdown and the limited interactions of the staff, the more staff in close contact with the residents the higher the risk of transmission. This reflected an increased risk of introducing the virus in the NH and an increase of contacts between residents and different staff members thus increasing their potential sources of contamination. This is supported by a modelling work conducted by Rosello et al., where importation of SARS-CoV-2 by staff, from the community, was found as the main driver of outbreaks [26]. A cohort study of French NHs during the COVID-19 pandemic, had lower mortality rates among NHs that implemented staff confinement with residents compared with those in a national survey [27]. These findings suggest that self-confinement of staff members with residents may help protect NHs residents from mortality related to COVID-19 and residents and staff from COVID-19 infection. In our study, we were unable to explore the effectiveness of the visitor bans measure because all NHs had implemented it. A literature review on the implementation of preventive and control measures reminds us that for this type of ban measures, beyond their potential effectiveness, the question of reintroducing visits also arises, particularly due to the negative impact of these restrictions on the cognitive and psychological well-being of residents [28]. This has been particularly highlighted in the German context by Koopmans et al. who have shown that facilities were sometimes still struggling to find the right balance between infection control and well-being [29, 30].

In the same way, Alzheimer’s units were conversely associated with mortality, suggesting that small living units for residents, with specific staff dedicated, would be in favor of a better control of the epidemic within the NH. It could guide us towards the reorganization of NHs with smaller units with attached staff instead of big units with flying staff. This is consistent with the idea that containment and cluster strategy are important to reduce the spread of COVID-19 outbreak in NHs [31]. In a study of 27 French NHs from a private group conducted after the first wave of COVID-19 that explored risk factors for infection, residents living in protected units due to behavioral disorders, had been more often contaminated [12]. This suggests that small units could slow the virus progression, but when a unit gets contaminated COVID-19 transmission is facilitated inside this unit, and emphasizes the importance for constant staff awareness and training regarding barrier measures.

However, due to some limitations, the results should be taken with caution. The study was conducted in a private network of NHs, which is not entirely representative of all the NHs in France and does not make it possible to identify if NHs were located in a rural or urban area. However, the sample covered the entire metropolitan area with a high participating rate and an important number of NHs investigated. In addition, data considered in this study were not longitudinal and did not allow us to study the chronology of the events. The low availability of screening tests (RT-PCR test) at the beginning of the first epidemic wave, made it difficult to count the “COVID-19” deaths. In addition, our study could not explore NH crowding, yet, in a cohort of Canadian NHs, crowding was common and crowded homes were more likely to experience larger and deadlier COVID-19 outbreaks [11]. Finally, the size of the nursing home as an effect modifier could not be taken into account due to an insufficient sample size.

## Conclusions

In this study carried out in a large French network of NHs where the control measures were largely implemented, we documented the role of the local fist-wave epidemic magnitude in the burden of COVID-19. At the NH level, results suggested that the organization in “small units” may have limited the impact of epidemic. These findings may help to improve the epidemic preparedness of NHs in the context of an exceptional health situation.

## Electronic supplementary material

Below is the link to the electronic supplementary material.

Supplementary Material 1: Survey questionnaire translated from French

## Data Availability

The data supporting the results of this study are available from the “Korian Foundation for the Ageing Well” but restrictions apply to the availability of these data, which for reasons of confidentiality are not publicly available. However, the data are available from the authors upon reasonable request and with permission from “Korian Foundation for the Ageing Well”. To request the data, you can ask to Biné Mariam Ndiongue, sending an email to bine-mariam.ndiongue@korian.fr.

## List of Abbreviations

NH: Nursing home
COVID-19: Corona virus disease
aOR: Adjusted odds ratio
SARS-CoV: Severe acute respiratory syndrome coronavirus
RT-PCR: Reverse Transcriptase Polymerase chain reaction
INSEE: National Institute of Statistics and Economic Studies
GMP: Groupe Iso-Ressource moyen pondéré
PMP: Pathos moyen pondéré
AIC: Akaike criterion information
CI: Confidence Interval
