# Supplementary material for: Impact of the first COVID-19 epidemic wave in a large French network of nursing homes: a cross-sectional study
Source: BMC Geriatr. 2023 Jul 3;23:406. doi: 10.1186/s12877-023-04078-8 (PMC10318739; doi:10.1186/s12877-023-04078-8)
Supplement: Supplementary file 1 — Supplementary Material 1: Survey questionnaire translated from French [file 12877_2023_4078_MOESM1_ESM.pdf]

Structure questionnaire

- Nursing home Name: ____
- *City:* ____
- *Post-code :* |___|___| |___|___||___|

Description of staff and care load

*Remarks : These answers relate to the institution **in its pre-crisis situation** COVID*

- NURSING HOME capacity: number of beds: ______
- Of which |___|___| 1-bed rooms, |___|____|2-bed rooms, |___|____| 3-bed rooms and more |____|____|
- Total NURSING HOME area: ______ m² of which:
  - Collective: ____m ²
  - Privative: _____ m²
- Number of buildings: |___|
- Air conditioning in the NURSING HOME: ☐ Yes ☐ No
  - If Yes, type of air conditioning (check the corresponding box):
    - Individual air conditioners ☐
    - Collective air conditioning with air handling unit ☐
    - Fan coil without air treatment ☐
- Last Weighted Average Pathos (PMP) validated by the RSO: _______
- Weighted Average IRM (GMP): _______
- Approximate proportion of residents walking:
  - < 10% ☐
  - 10-20% ☐
  - 20-30% ☐
  - 30-40% ☐
  - >50% ☐
- Approximate proportion of falling residents:
  - < 10% ☐
  - 10-20% ☐
  - 20-30% ☐
  - 30-40% ☐
  - >50% ☐
- Average age of residents: _______
- Mobile Palliative Care Team intervention over the past 12 months:

☐Yes, signed agreement YES/NO
☐No

Description of living, care and technical spaces:

		Does the institution have following spaces?	
Units of life in the floors	Alzheimer's Unit/Space	o Yes Number:	o No
	FSEP/ Day Greeting	o Yes Number:	o No
	Independent living units (entrance/ access independent)	o Yes Number:	o No
	Floor lounges	o Yes Number:	o No
	Dining room on the floors	o Yes Number:	o No
Spaces of life common to the ground floor pavement	A common dining room	o Yes	o No
	Living room(s) on the ground floor	o Yes Number:	o No
	Space(s) for activities, animations, the shows	o Yes Number:	o No
	Dedicated television area(s)	o Yes Number:	o No
	Salon(s) de coiffure/ esthétique	o Yes Number:	o No
	Exterior space(s) fitted out	o Yes Number:	o No
Spaces of care shared	A space of physiotherapy/ rehabilitation	o Yes	o No
	A space for medical care	o Yes	o No
	Presence of a system suitable for weighing (balance chair, coupled weighing system in the morning)	o Yes	o No
	A multi-sensory room (Snoezelen type, balneotherapy, wellness, etc.)	o Yes	o No
	At least 2 elevators	o Yes	o No

Human Resources and Organization Description since January 1, 2020

- Physician Coordinator in place and still present on March 1' 2020:
  - Yes
    If the position is held by multiple physicians, number of people |___|____|
  - No

- Nurse Coordinator in place and still present on March 1, 2020: yes no
- Volunteers
  Number of people |___|___|
- Total number of people (all employees + volunteers): |___|____|_____|
- Number of prescribers |___|___|

Access to hygiene and training expertise:

Is your establishment accompanied by an external hygiene team?

- yes
- no

If yes: this support was in place before the Covid epidemic: yes no In 2020 in previous years

If yes: did this coaching also take place during the Covid-19 outbreak yes no

Type of support (training/audits/etc.):
____
____

Has training of the health professionals of the establishment been carried out on standard precautions in the last 2 years?: yes no

At least one hygiene correspondent is identified among NURSING HOME staff.

- yes
- no
- don't know

Description of measures implemented since February [1], 2020

Number of residents present as of March 1: |___|____|____|
Totally dependent (GIR 1-2): |___|____|_____|
Partially dependent (IRM 3-4): |___|____|_____|
Standalone (IRM 5-6): |___|____|_____|

Stopping visits to the establishment: ○ yes ○ no
Date of implementation: ___/____/_______
Recovery Date: ___/____/_______

Closing of day receptions: ○ yes ○ no ○ Not concerned
Date of implementation: ___/____/_______
Date of re-opening: ___/____/_______

Individual confinement of residents in rooms: ○ yes ○ no
Date of implementation: ___/____/_______
Stop Date: ___/____/_______

Sectoring: ○ yes ○ no
Date of implementation: ___/____/_______
Stop Date: ___/____/_______

One or more Covid units have been dedicated to Covid residents: ○ yes ○ no

If yes: staff dedicated to this unit during the day? ○ yes ○ no

If yes: was staff dedicated to this unit at night? ○ yes ○ no

Have some units taken in both positive COVID residents and other residents?
○ yes
○ no

Training on standard and additional precautions has been carried out: ○ yes ○ no
if yes by whom?: ____

Practice audits were conducted: ○ yes ○ no
if yes by whom?: ____

Covid Case Descriptions

For these questions, the period to be considered is from 01/02/2020 to 15/06/2020

Have you had COVID cases: ○ YES ○ NO

- No. of resident cases
- No. of employee cases
- Resident and employee mortality

If YES, Date of first positive test among residents: ___/____/_______

- Were COVID cases initially housed in the same unit? ○ YES ○ NO
  - If YES: was this unit an ALzheimer space? ○ YES ○ NO

Existence of an epidemic outbreak in the vicinity of the institution (in the living area) preceding or concomitant with the 1st case in the institution: yes ○ no ○

If yes, case knowledge contacts among employees/ residents/ families related to this epidemic outbreak: ○ yes ○ no
